# Vestibular Dysfunctions in Sudden Sensorineural Hearing Loss: A Systematic Review and Meta-analysis

**DOI:** 10.3389/fneur.2018.00045

**Published:** 2018-02-05

**Authors:** Huiqian Yu, Huawei Li

**Affiliations:** ^1^Otorhinolaryngology Department, ENT Institute, Affiliated Eye and ENT Hospital of Fudan University, Shanghai, China; ^2^Institutes of Biomedical Sciences, Fudan University, Shanghai, China

**Keywords:** vestibular dysfunction, vertigo, sudden sensorineural hearing loss, caloric test, cervical vestibular-evoked myogenic potential, ocular vestibular-evoked myogenic potential, meta-analysis

## Abstract

**Background:**

Sudden sensorineural hearing loss (SSHL) not only involves cochlear function but might also be accompanied by vestibular disturbances. The assessment of vestibular function could be of great relevance in SSHL.

**Objective:**

To investigate the prevalence of vestibulocochlear lesions in SSHL and the correlation of specific vestibular organs with hearing prognosis.

**Data sources:**

A complete literature search of eligible studies in the PubMed and EMBASE databases was performed.

**Study selection:**

For our aim, studies that focused on vestibular examination in the case of SSHL were retrieved, including caloric tests, cervical vestibular-evoked myogenic potential (cVEMP) tests, or ocular vestibular-evoked myogenic potential (oVEMP) tests.

**Results:**

Of the 18 studies included, a caloric test was performed in 16 studies, cVEMP in 13 studies, and oVEMP in 5 studies, and together the studies included a total population of 1,468 subjects. The scores on the Agency for Healthcare Research and Quality (AHRQ) questionnaire ranged from 6 to 11. These results indicated that the most commonly damaged vestibular organ in SSHL was the utricle and superior vestibular pathway (U + S) followed by the lateral semicircular canal and superior vestibular pathway (LSC + S), the saccule and inferior vestibular pathway (S + I), and the cochlea only (C only). The meta-analysis indicated that SSHL patients with vertigo have a statistically increased risk of vestibular organ lesions compared with those without vertigo, including the LSC + S subgroup (OR = 4.89, 95% CI = 1.20–19.93, *I*^2^ = 80%, *p* = 0.03) and the S + I subgroup (OR = 3.58, 95% CI = 1.61–7.95, *I*^2^ = 0%, *p* = 0.002). The pooled possibility of hearing recovery within the LSC + S lesion group was less than half that of the non-LSC + S lesion group (OR = 0.24, 95% CI = 0.11–0.52, *I*^2^ = 68%, *p* = 0.0003).

**Conclusion:**

This study shows the relevance of vestibular damage concomitant with SSHL and that SSHL patients with vertigo are at an increased risk of vestibular organ lesions compared with patients without vertigo. LSC + S lesions thus appear to be a critical variable that influence the possibility of hearing improvement in SSHL.

## Introduction

Sudden sensorineural hearing loss (SSHL) is clinically characterized by a rapid onset sensorineural hearing loss of more than 30 dB for at least three contiguous audiometric frequencies within a period of 3 days (1–3). Approximately 5–20 per 100,000 persons are afflicted with SSHL annually, and nearly 20–60% of SSHL patients complain of simultaneous vertigo (4–6). Because of the close correlation between the cochlea and the vestibular organs both anatomically and phylogenetically, impairment of cochlear function, which can result in SSHL, might be accompanied by vestibular disturbances (7, 8). Thus, the pathology of SSHL involves not only the cochlea but also, at least to some extent, the vestibular organs (7–9).

Recently, some vestibular diagnostic methods have been included in the general evaluation of patients suffering from SSHL, such as the caloric test, cervical vestibular-evoked myogenic potential (cVEMP), and ocular vestibular-evoked myogenic potential (oVEMP). The caloric test is a method for investigating lateral semicircular canal function and superior vestibular integrity, the cVEMP can be used for assessing saccular function and the inferior vestibular pathway, and oVEMP can be used for evaluating utricular function and the superior vestibular pathway (10–13). Some clinical studies have been performed to identify the vestibular dysfunction in patients with SSHL. Abnormal caloric test results have been reported in 39–74% of patients with SSHL (14–16), and the results of Korres et al. and Niu et al. indicated that abnormal vestibular examinations are associated with profound hearing loss in SSHL patients (7, 17). Iwasaki et al. found that the saccule is more often involved than the semicircular canal in SSHL patients with vertigo (18); however, recent results by Lee et al. do not support the hypothesis above, and they found higher rates of lesions in the semicircular canal of SSHL patients (19). Thus, the vestibulocochlear lesion patterns, the clinical relevance of vertigo in relation to vestibulocochlear lesion location, and the correlation of vestibular organ damage with the prognosis of hearing loss in SSHL have not yet achieved consensus. To address this issue, we performed a systematic review and meta-analysis to shed light on the presumed pathomechanisms that are involved in the clinical manifestation and prognosis of SSHL.

## Materials and Methods

### Data Sources and Search Strategy

The initial literature review of relevant studies for assessing the correlation of lesion pattern with the clinical manifestation of SSHL, with or without the presence of vertigo, was carried out in the PubMed and EMBASE databases using a combination of keywords, including “sudden hearing loss,” “sudden deafness,” “caloric test,” “canal paresis,” “VEMP,” and “vestibular-evoked myogenic potential.” All pertinent articles or abstracts that were published in English were retrieved with no restrictions on the date of publication. Additional papers were searched for in the reference lists of the identified papers.

### Eligibility Criteria for Study Selection

The eligibility criteria for inclusion in our analysis were as follows: retrospective or prospective original research containing the routine examinations of vestibular function in SSHL patients; information on whether vestibular symptoms were involved in patients with SSHL; and a homogenous examination regime (caloric test, cVEMP, or oVEMP) in each group. Excluded papers included comments, reviews, case reports, practice guidelines, and editorials. Those studies that failed to provide sufficient information to calculate these variables were also excluded in our analysis.

### Data Abstraction

The required information and data were independently extracted from the selected studies and quantified by the two authors in a standardized manner. The characteristics of each study included year of publication, research design, age and number of participants, presence of vertigo, presence of canal paresis, examination regime (caloric test, cVEMP, or oVEMP), and hearing improvement, including the number of subjects with good or poor hearing recovery in subgroups of abnormal vs. normal caloric response, abnormal vs. normal cVEMP response, and abnormal vs. normal oVEMP response. The retrieved data were compared and revised in mutual agreement between the two authors.

### Quality Assessment and Sensitivity Analysis

The checklist of the Agency for Healthcare Research and Quality (AHRQ) was used to assess the quality and bias risk of the included studies (20). This scale has 11 evaluation criteria, including source of information, inclusion and exclusion criteria, time period, consecutive subjects, mask of subjective components, quality assurance, explanation of exclusions, control of confounding factors, method of handling missing data, completeness of data collection, and follow-up. An item is scored as 1 if included in the article and 0 if not. A score of 8 or more is indicative of a high-quality study.

A sensitivity analysis was further performed to investigate the heterogeneity of the included data and to estimate the impacts of each study on pooled outcomes based on the rule of omission. It also was used to assess and correct any publication bias. A study was confirmed as a source of heterogeneity if the *I*^2^ value decreased significantly.

### Statistical Analysis

The various studies included non-comparative data and comparative data in a dichotomous pattern. Non-comparative analyses were carried out using the R version 3.4.1 software (R Foundation for Statistical Computing). We estimated the cumulative prevalence (event rate) from each study and the corresponding 95% confidence interval (CI) for each outcome in the non-comparative data. Event rates for each investigation were pooled in the meta-analysis across eligible studies using a fixed-effect model (*p* > 0.1) or a random-effect model (*p* ≤ 0.1). Comparative analyses were performed using Review Manager (RevMan) software (Version 5.3; Copenhagen: The Nordic Cochrane Centre, the Cochrane Collaboration, 2014). The prognosis outcomes were displayed using 95% CI in forest plots. The pooled association between the presence of vertigo and vestibular lesions was quantitatively analyzed using the Mantel–Haenszel method with a combined estimate of the odds ratio (OR) (21). The heterogeneity of the included studies was statistically assessed using a *p*-value (heterogeneity was statistically significant when *p* ≤ 0.05) and an *I*^2^ statistic (a derivative of the Cochran Q statistic, *I*^2^ < 50% indicates low heterogeneity and *I*^2^ > 50% indicates high heterogeneity) (22, 23). A fixed-effect model was adopted when *I*^2^ < 50%; otherwise, a random-effect model was adopted. The result was considered to be statistically significant when *p* ≤ 0.05, and the potential asymmetry and publication bias was visually estimated from funnel plots.

## Results

### The Characteristics and Quality Assessments of the Included Studies

**Figure 1:**
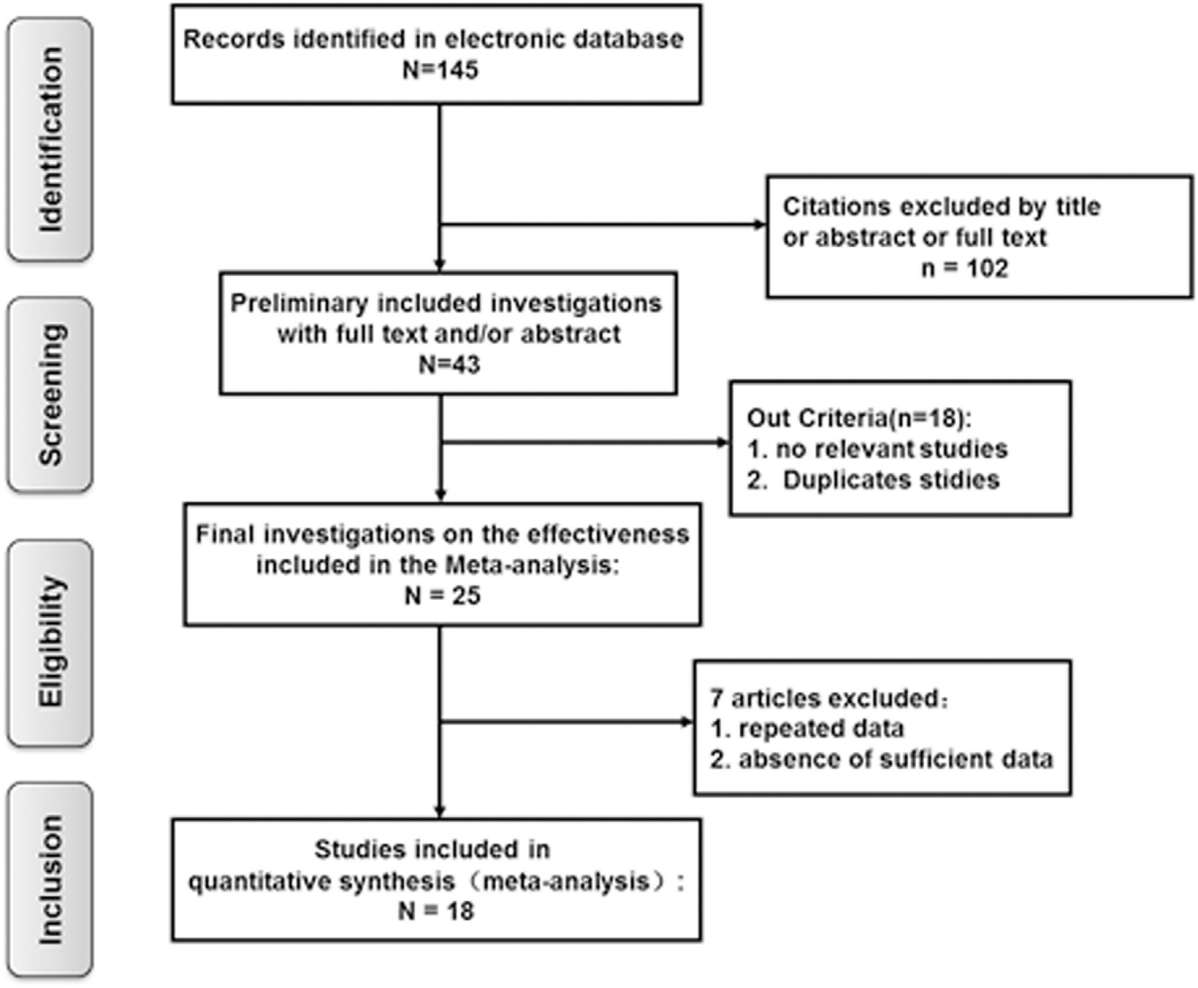
Flow chart of the study selection for the meta-analysis.

Based on the criteria mentioned above, 145 potentially relevant references were initially identified. Of these, 102 studies were excluded after screening by the title, abstract, or full text. A further 18 studies were removed because they lacked vestibular function examination in SSHL with or without vestibular symptoms. Of the remaining 25 investigations, 7 articles were excluded from the final analysis due to duplicated data containing the same participants or to not providing sufficient information to calculate these variables. Finally, a total of 18 eligible articles met our inclusion criteria and were included in the final meta-analysis (7, 8, 14, 17–19, 24–35) (Figure 1).

The main demographic features and clinical characteristics of the patients are listed in Table 1. Of the 18 studies included, 15 were retrospective studies (7, 14, 17–19, 24–33), 2 were prospective studies (8, 35), and 1 was a prospective/retrospective study (34), and the studies covered a total population of 1,468 subjects. The mean age ranged from 44 to 62 years, with the proportion of females ranging from 35 to 74%. The occurrence rate of vertigo was also summarized as a list. Concerning the types of vestibular examinations, the caloric test was performed in 16 studies (7, 8, 14, 17–19, 24–33), the cVEMP test was performedin 13 studies (7, 8, 14, 17–19, 24–28, 34, 35), and the oVEMP test was performed in 5 studies (17, 24, 26, 27, 34). In all 18 investigations, hearing recovery for abnormal and normal caloric response was provided in 7 studies (18, 19, 27, 29–31, 33), the number of patients with hearing improvement with abnormal and normal cVEMP response was provided in 4 studies (18, 19, 25, 27), and 1 study provided hearing outcomes for abnormal and normal oVEMP responses (27). The AHRQ scores ranged from 6 to 11, including 15 high-quality studies and 3 medium-quality studies (Table 2).

**Table 1:** Literature reports of selected studies.

Study	Research type	Subject	Age	Vertigo (Y/N)	Criteria of canal paresis	Location of lesion			
						LSC + S (%)	S + I (%)	U + S (%)	C only (%)
Liu et al. (24)	R	35	–	21/14	>25%	19 (54)	17 (49)	22 (63)	–
Lee et al. (19)	R	92	51	52/40	>20%	46 (50)	29 (32)	–	35 (38)
Chen and Young (25)	R	14	48	5/9	>25%	9 (64)	3 (21)	–	–
Fujimoto et al. (26)	R	23	62	23/0	>20%	12 (52)	15 (65)	10 (43)	6 (23)
Stamatiou et al. (14)	R	86	51	32/54	>22%	45 (52)	26 (30)	–	36 (42)
You et al. (27)	R	75	54	47/28	>25%	28 (37)	35 (47)	36 (48)	–
Iwasaki et al. (18)	R	22	54	22/0	>20%	10 (45)	17 (77)	–	4 (18)
Oiticica et al. (28)	R	21	52	–	No data	11 (58)	5 (36)	–	5 (29)
Niu et al. (17)	R	149	44	87/62	≥25%	68 (73)	73 (49)	84 (56)	–
Ryu et al (8)	P	35	48	17/18	>22%	19 (54)	19 (54)	–	–
Korres et al. (7)	R	104	53	36/68	>22%	52 (50)	30 (29)	–	45 (43)
Pogson et al. (34)	R/P	27	57	27/0	–	–	9 (33)	11 (41)	–
Hong et al. (35)	P	52	55	0/52	–	–	14 (27)		–
Weiss et al. (29)	R	167	54	–	No data	29 (17)	–	–	–
Kim et al. (30)	R	90	–	90/0	≥25%	38 (42)	–	–	–
Lee and Ban (31)	R	298	48	–	No data	48 (16)	–	–	–
Kim (32)	R	43	52	43/0	≥25%	17 (40)	–	–	–
Shih et al. (33)	R	135	51	57/78	>20%	39 (29)	–	–	–

*Literature reports of the selected studies. R, retrospective; P, prospective; Y/N, yes/no; LSC + S, lateral semicircular canal and superior vestibular nerve; S + I, saccule and inferior vestibular nerve; U + S, utricle and superior vestibular nerve; C only, cochlear only*.

**Table 2:** Quality control of the selected studies according to the criteria of the Agency for Healthcare Research and Quality (AHRQ).

Study	Quality assessment by AHRQ											

	1	2	3	4	5	6	7	8	9	10	11	Score
Liu et al. (24)	★	★	★	★	○	★	★	★	○	★	○	8
Lee et al. (19)	★	★	★	★	★	★	○	★	○	★	○	8
Chen and Young (25)	★	★	★	○	○	○	○	★	○	★	★	6
Fujimoto et al. (26)	★	★	★	○	★	★	○	★	○	★	○	7
Stamatiou et al. (14)	★	★	★	○	★	★	★	★	★	★	○	9
You et al. (27)	★	★	★	★	○	★	★	★	★	★	○	9
Iwasaki et al. (18)	★	★	★	★	○	★	★	★	○	★	★	9
Oiticica et al. (28)	★	★	★	★	○	★	★	★	○	★	★	9
Niu et al. (17)	★	★	★	★	○	★	★	★	○	★	○	8
Ryu et al. (8)	★	★	★	★	★	★	★	★	★	★	★	11
Korres et al. (7)	★	★	★	★	★	★	★	★	★	★	★	11
Pogson et al. (34)	★	★	★	★	★	★	★	★	○	★	★	11
Hong et al. (35)	★	★	★	○	○	★	★	★	★	★	★	9
Weiss et al. (29)	★	★	★	★	○	★	★	★	○	★	○	8
Kim (30)	★	★	★	★	○	★	★	★	○	○	○	7
Lee and Ban (31)	★	★	★	★	★	★	★	★	★	★	★	11
Kim (32)	★	★	★	★	○	★	★	★	★	★	○	9
Shih et al. (33)	★	★	★	★	★	★	★	★	★	○	★	10

*1, source of information; 2, inclusion and exclusion criteria; 3, time period; 4, consecutive subjects; 5, mask of subjective components; 6, quality assurance; 7, explanation of exclusions; 8, control of confounding factors; 9, handling of missing data; 10, completeness of data collection; 11, follow-up. ★, present and score = 1; ○, not present or unclear and score = 0*.

### The Prevalence of Vestibulocochlear Lesion Patterns in Sudden Sensorineural Hearing Loss (SSHL)

**Figure 2:**
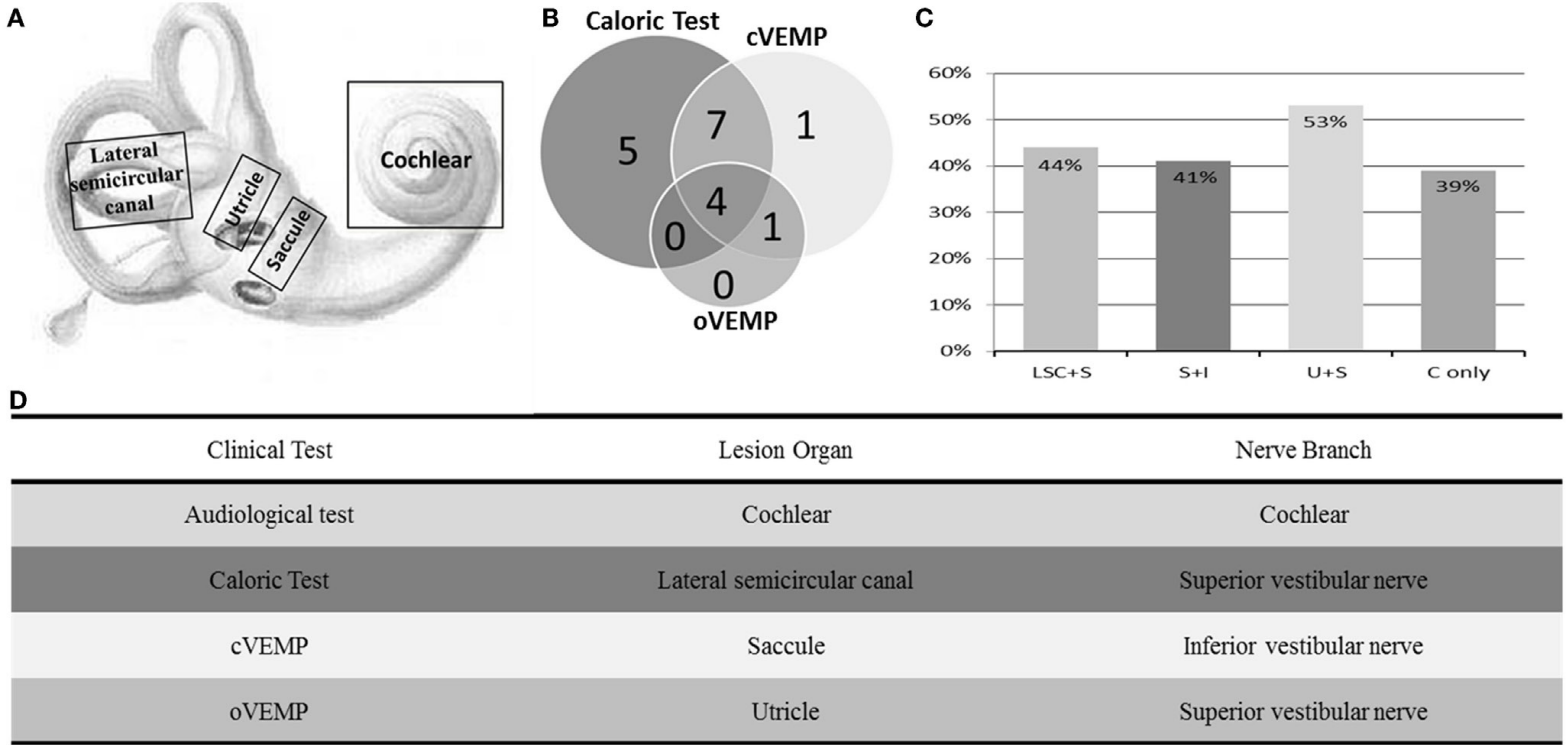
Summary of audio-vestibular abnormalities in SSHL. **(A)** Schematic diagram of the vestibulocochlear lesion patterns, including the cochlea, saccule, utricle, and lateral semicircular canal. **(B)** Distributions of vestibular test abnormalities of all 18 studies, including 16 by caloric test, 13 by cervical vestibular-evoked myogenic potential test (cVEMP), and 5 by ocular vestibular-evoked myogenic potential test (oVEMP). **(C)** Percentage of inner ear lesion locations in the included studies––LSC + S, abnormal caloric test; S + I, abnormal cVEMP test; U + S, abnormal oVEMP test; and C only, cochlear impairment only. **(D)** Table of clinical tests performed, organs involved, and their corresponding innervation.

**Figure 3:**
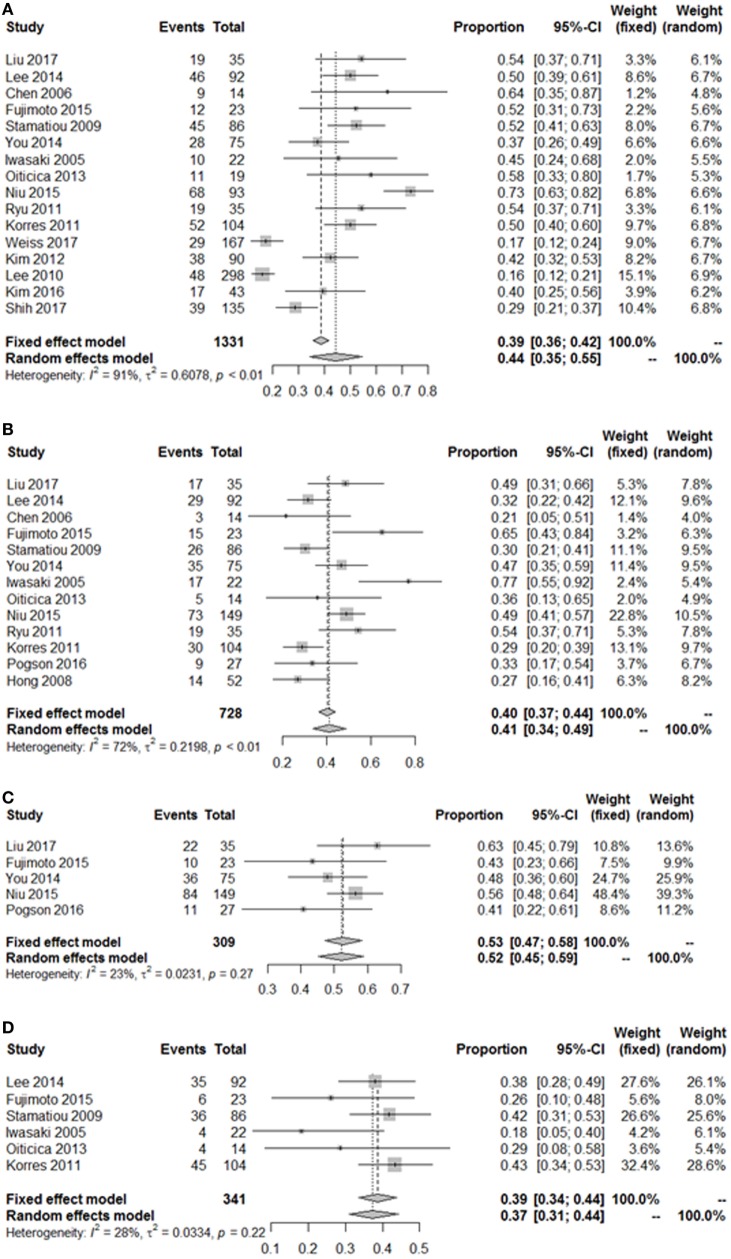
Pooled event rate of inner ear organ lesion locations. **(A)** Pooled occurrence rate of lateral semicircular canal lesion and superior vestibular nerve (LSC + S). **(B)** Pooled occurrence rate of saccule and inferior vestibular nerve lesion (S + I). **(C)** Pooled occurrence rate of utricle and superior vestibular nerve lesion (U + S). **(D)** Pooled occurrence rate of cochlea-only lesion (C only). Random, random-effect method; fixed, fixed-effect method.

**Figure 4:**
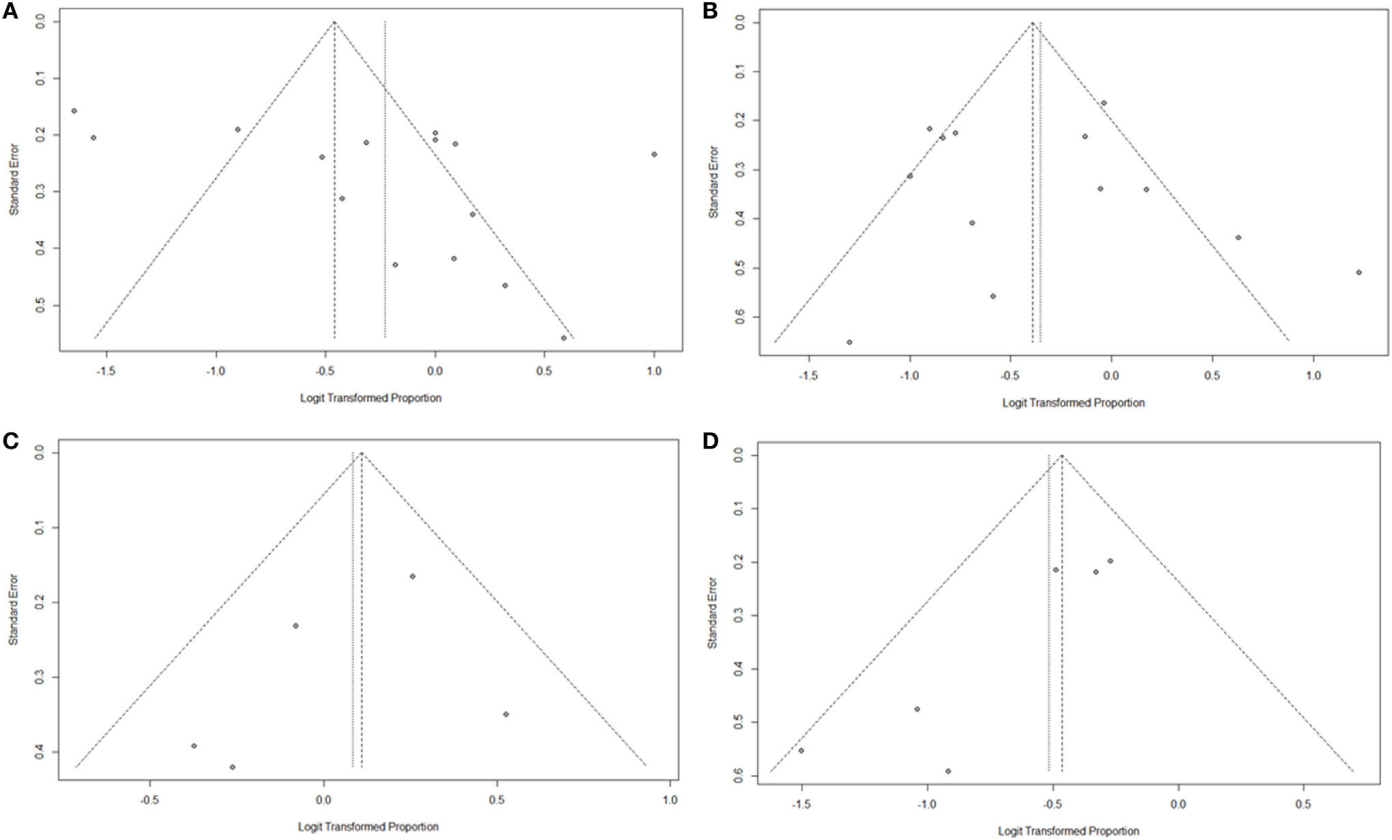
Funnel plots for the evaluation of publication bias. **(A)** Funnel plot of the included studies in the meta-analysis of pooled proportions within the LSC + S subgroup. **(B)** Funnel plot of the included studies in the meta-analysis of pooled proportions within the S + I subgroup. **(C)** Funnel plot of the included studies in the meta-analysis of pooled proportions within the U + S subgroup. **(D)** Funnel plot of the included studies in the meta-analysis of pooled proportions within the C only subgroup.

As summarized in Figure 2, lesions of the lateral semicircular canal and superior vestibular pathway (LSC + S) were evaluated by the caloric test; lesions of the saccule and inferior vestibular pathway (S + I) were evaluated by the cVEMP test; lesions of the utricle and/or superior vestibular pathway (U + S) were evaluated by the oVEMP test, and the remaining lesions were assigned as cochlear lesions only (C only). We performed a meta-analysis of the prevalence of inner ear damage within the LSC + S, S + I, U + S, and C only subgroups of SSHL independently. There was a combined proportion of 0.44 (95% CI, 0.34–0.55, *p* < 0.01) with a random-effect model (*I*^2^ = 91%) in the mean occurrence rate of LSC + S; a combined proportion of 0.41 (95% CI, 0.34–0.49, *p* < 0.01) with a random-effect model (*I*^2^ = 74%) in the mean occurrence rate of S + I; a combined proportion of 0.53 (95% CI, 0.47–0.58, *p* = 0.27) with a fixed-effect model (*I*^2^ = 23%) in the mean occurrence rate of U + S; and a combined proportion of 0.39 (95% CI, 0.34–0.44, *p* = 0.22) with a fixed-effect model (*I*^2^ = 39%) in the mean occurrence rate of C only. Forest plots of the success rates are shown in Figure 3. Asymmetry was observed in the pool of data from the included investigations, indicating that there was some publication bias, as demonstrated by a funnel plot (Figure 4). The results indicated that the prevalence of inner ear organ damage was greatest in the U + S subgroup in SSHL, followed by LSC + S, S + I, and C only.

Considering the bias of vestibular balance in the studies, we performed further meta-analyses within the vertigo subgroup and the non-vertigo subgroup (Table 3). The pooled proportions for LSC + S, S + I, U + S, and C only within the vertigo subgroup were 0.50 (95% CI, 0.44–0.56, *I*^2^ = 44%, *p* = 0.1), 0.57 (95% CI, 0.41–0.72, *I*^2^ = 70%, *p* = 0.01), 0.54 (95% CI, 0.31–0.77, *I*^2^ = 77%, *p* = 0.01), and 0.25 (95% CI, 0.16–0.35, *I*^2^ = 0%, *p* = 0.53), respectively. The mean occurrence rates of LSC + S and S + I in the non-vertigo subgroup were 0.29 (95% CI, 0.00–0.58, *I*^2^ = 93%, *p* < 0.01) and 0.23 (95% CI, 0.16–0.31, *I*^2^ = 5%, *p* = 0.35), respectively. We were unable to obtain the pooled proportion in the U + S and C only subgroups because there was only one study including these subgroups. From the data above, we found that more than half of the patients suffered from some form of vestibular organ lesion in SSHL with vertigo, while only a quarter of the patients had damage of the vestibular organ in SSHL without vertigo.

**Table 3:** Summary of the meta-analysis for the prevalence of the vestibulocochlear lesion patterns in SSHL.

Location of lesion	Stratification	Included studies	Proportion (95% CI)	Heterogeneity	
				*I*^2^	*P*-value
LSC + S	Overall	16	0.44 [0.34, 0.55]	91%	<0.01
	With vertigo	7	0.50 [0.44, 0.56]	44%	0.1
	Without vertigo	3	0.29 [−0.00, 0.58]	93%	<0.01

S + I	Overall	13	0.41 [0.34, 0.49]	72%	<0.01
	With vertigo	5	0.57 [0.41, 0.72]	70%	0.01
	Without vertigo	3	0.23 [0.16, 0.31]	5%	0.35

U + S	Overall	5	0.53 [0.47, 0.58]	23%	0.27
	With vertigo	3	0.54 [0.31, 0.77]	77%	0.01
	Without vertigo	1	–	–	–

C only	Overall	6	0.39 [0.34, 0.44]	28%	0.22
	With vertigo	3	0.25 [0.16, 0.35]	0%	0.53
	Without vertigo	1	–	–	–

### The Clinical Value of Vertigo as a Prognostic Indicator of Lesion Location in SSHL

**Figure 5:**
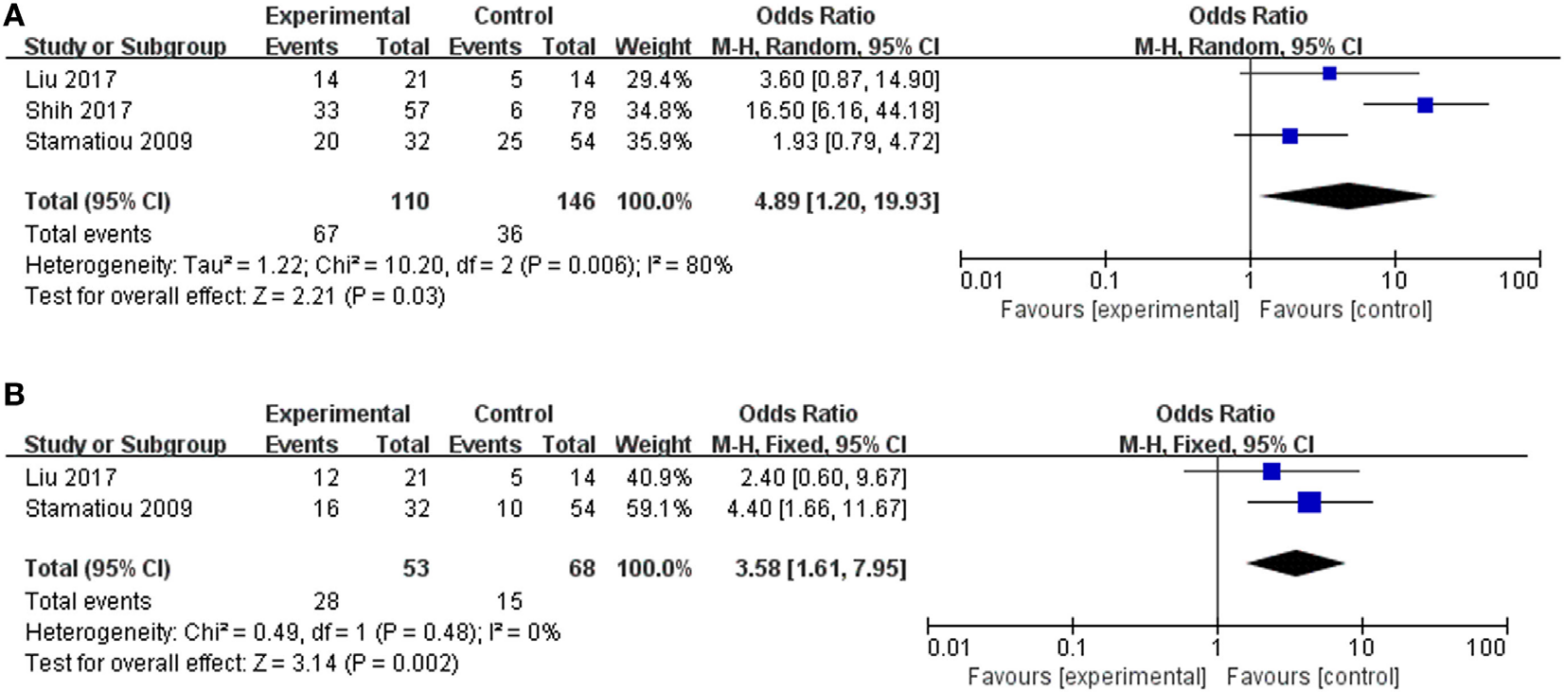
Forest plots of the synthesized data from the selected studies. **(A)** Comparison of the occurrence rate of lateral semicircular canal and superior vestibular nerve lesion (LSC + S) between the vertigo and non-vertigo groups. **(B)** Comparison of the occurrence rate of saccule and inferior vestibular nerve lesion (S + I) between the vertigo and non-vertigo groups. OR, odds ratio; M–H, Mantel–Haenszel method; random, random-effect method; fixed, fixed-effect method.

The clinical value of vertigo in inner ear organ damage was assessed by meta-analysis. Three studies described the occurrence rates of LSC + S lesions in a dichotomous pattern (vertigo group vs. non-vertigo group). We adopted a random-effect model with a high heterogeneity (*I*^2^ = 80%) to evaluate whether LSC + S in the vertigo group was more likely to be damaged than in the non-vertigo group. The weighted mean OR was 4.89, and the 95% CI was 1.20–19.93 (Figure 5A) with statistical significance (*p* = 0.03).

**Figure 6:**
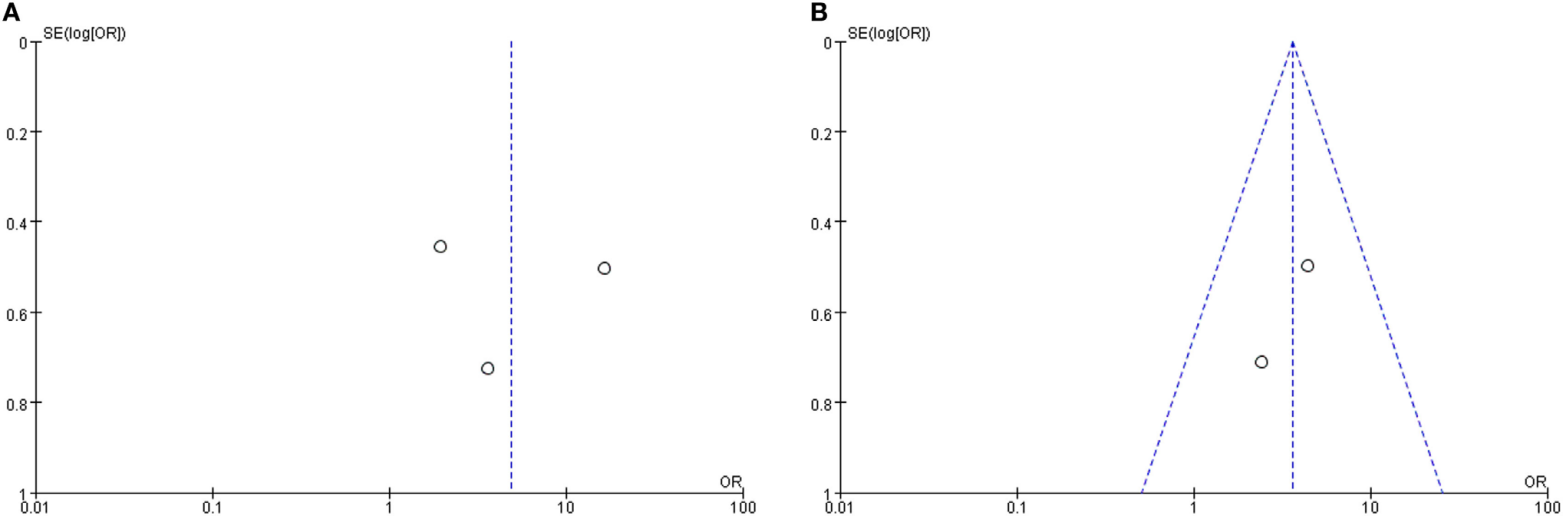
Funnel plots for the evaluation of publication bias. **(A)** Funnel plot of the included studies in the meta-analysis of the occurrence rate of lateral semicircular canal and superior vestibular nerve lesion (LSC + S). **(B)** Funnel plot of the included studies in the meta-analysis of the occurrence rate of saccule and inferior vestibular nerve lesion (S + I). MD, mean difference; OR, odds ratio.

Only two studies were assessed in the S + I subgroup, and there was a combined OR of 3.58 (95% CI 1.61–7.95, *p* = 0.002) with a fixed-effect model (*I*^2^ = 0%) (Figure 5B). There was a statistically significant difference in the occurrence rates of LSC + S and S + I lesions between the vertigo and non-vertigo groups, which suggests that the presence of vertigo is indicative of a positive prognosis of greater vestibular dysfunction. Asymmetry was demonstrated by a funnel plot (Figure 6).

### The Correlation of Vestibular Organ Damage with Prognosis of Hearing Variables in SSHL

**Figure 7:**
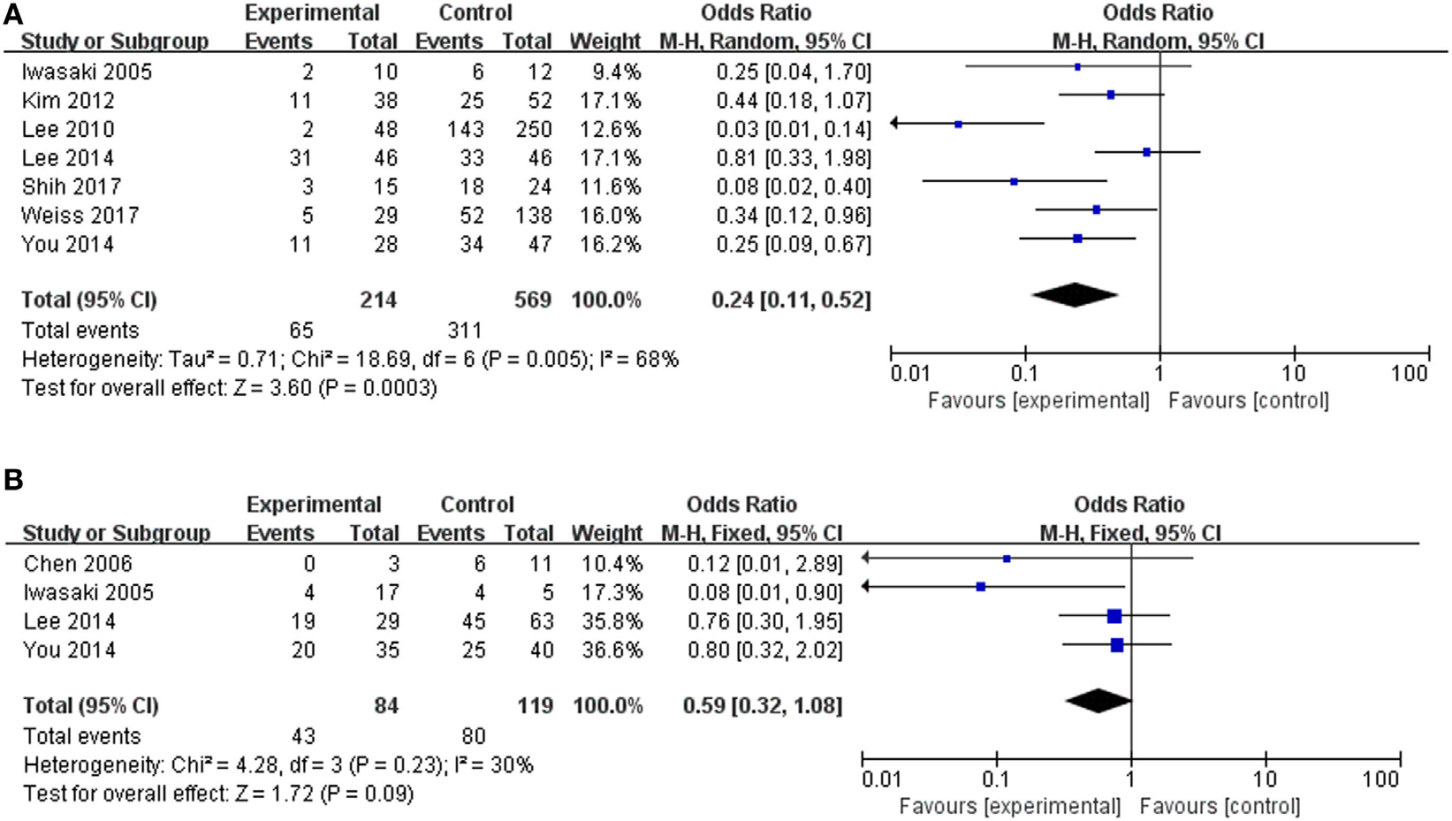
Forest plots of synthesized data from the selected studies. **(A)** Comparison of hearing improvement between the abnormal caloric response and normal caloric response groups. **(B)** Comparison of hearing improvement between the abnormal cVEMP response and normal cVEMP response groups. OR, odds ratio; M–H, Mantel–Haenszel method; random, random-effect method; fixed, fixed-effect method.

We next evaluated the hearing recovery of SSHL with or without LSC + S lesions by the caloric test in a total of 7 studies. We adopted a random-effect model with a high heterogeneity (*I*^2^ = 68%) to evaluate whether LSC + S lesions were more likely to be associated with poor hearing recovery compared with the group without LSC + S lesions. The weighted mean OR was 0.24, and the 95% CI was 0.11–0.52 (Figure 7A) with statistical significance (*p* = 0.0003).

**Figure 8:**
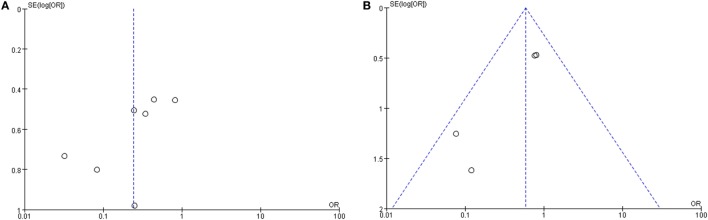
Funnel plots for the evaluation of publication bias. **(A)** Funnel plot of the included studies in the meta-analysis of hearing improvement between the abnormal caloric response and normal caloric response groups. **(B)** Funnel plot of the included studies of hearing recovery rate between the abnormal cVEMP response and normal cVEMP response groups. MD, mean difference; OR, odds ratio.

The clinical value of S + I damage according to the cVEMP test was assessed by meta-analysis. Four studies described the hearing outcomes of SSHL with or without S + I lesion. There was a combined OR of 0.59 (95% CI 0.32–1.08, *p* = 0.09) with a fixed-effect model (*I*^2^ = 30%) (Figure 7B). Asymmetry was demonstrated by a funnel plot (Figure 8). Due to a lack of data, we were unable to estimate the role of U + S lesions by oVEMP in the prognosis of hearing outcome in SSHL.

### Sensitivity Assessment and Publication Bias

**Figure 9:**
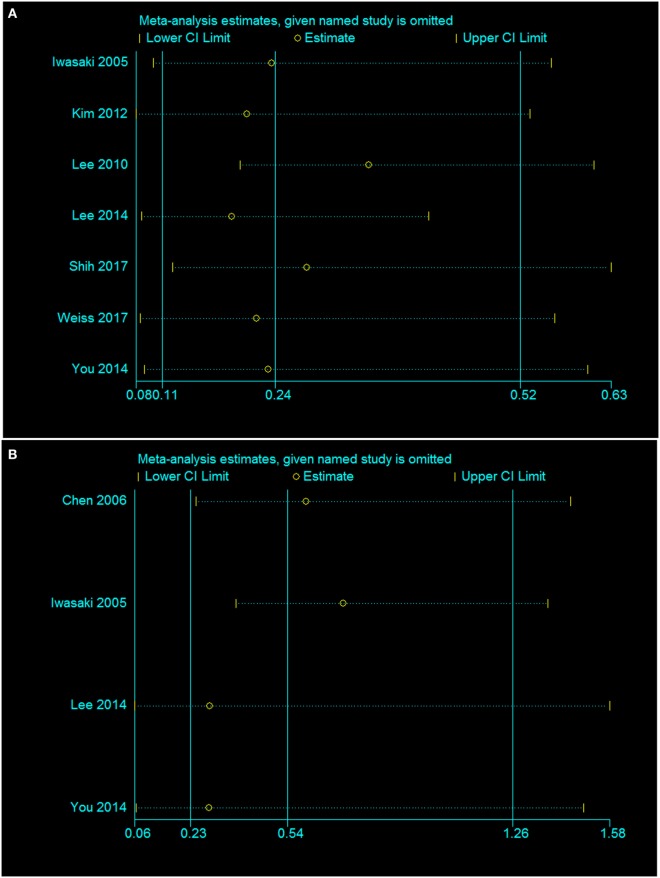
Sensitivity analysis of the association of the presence or absence of caloric response **(A)** and cVEMP response **(B)** in the hearing recovery rate in SSHL.

Finally, a sensitivity analysis was performed to assess and correct the publication bias based on the rule of omission. When each study was excluded sequentially to assess the stability of the final results, we found that no investigation affected the pooled risk estimate (Figure 9). However, we did find that the heterogeneity was significantly reduced if the investigation by Shih et al. was omitted in our study. In that case, the funnel plot was symmetrical in general and the heterogeneity test changed from *I*^2^ = 80%, *p* = 0.03 to *I*^2^ = 0%, *p* = 0.03.

## Discussion

Vestibular involvement in SSHL was first reported in 1949, and the role of vertigo in the clinical manifestation and prognosis of SSHL has been extensively studied since then (36). A number of studies have revealed the prevalence of inner ear organ lesion location in SSHL with or without vertigo (14–16, 18). However, some findings about the exact role of vestibular dysfunctions and lesion locations in SSHL have been unclear or contradictory, which led us to perform the present meta-analysis in order to draw proper conclusions with regard to the available evidence.

In the present systematic review and meta-analysis, we first found that vestibular organs were involved in SSHL regardless of the presence of vertigo, which indicated that vertigo might not be an independent and determining factor in SSHL. This phenomenon might instead be the result of the close correlation of the cochlear and vestibular organs both anatomically and genetically (7, 8).

To further evaluate the extent to which the entire inner ear, especially the vestibular organs, is involved in SSHL, we quantified the prevalence of the vestibulocochlear lesion patterns in our meta-analysis. The pooled event rates in our review showed that the U + S were the most susceptible to damage in SSHL, followed by the LSC + S and the S + I. Anatomically, the longer and narrower bony canal makes the superior vestibular nerve more susceptible to possible ischemic labyrinthine changes or other entrapments compared with the inferior vestibular nerve or singular nerves (37, 38). Manzari et al. also found that impairment of the utricle alone, as demonstrated by the results of the oVEMP test, was commonly associated with vestibular symptoms in the SSHL patients (39). Similarly, a significantly declining trend of inner ear deficits from the utricle to the saccule, and semicircular canals was identified after exposure to organic solvents (40). Peng-Chieh et al. explained that it was a combined result of peripheral and central parts of auditory pathways (40). Thus, the pathogenesis and pathological changes involved in otolith organ dysfunction in SSHL should be further explored in future studies.

In addition, our analysis also suggested that SSHL patients with vertigo are at an increased risk of vestibular organ lesions compared with patients who are not affected by vertigo. This result indicated that vertigo might be an important prognostic indicator in vestibulocochlear lesion patterns in SSHL. Furthermore, consistent results were confirmed by the statistical significance in the comparative dichotomous data in the LSC + S and S + I subgroups, which supports the above conclusions.

To determine the role of damage to the vestibular apparatus in the prognosis of hearing outcomes in SSHL, we quantified the occurrence of hearing improvement in our meta-analysis. We found that the likelihood of hearing recovery in the abnormal caloric response group was significantly worse than in the normal caloric response group, which might indicate that the abnormal caloric response is a negative prognostic marker in SSHL. In support of the hypothesis of Kim et al. the presence of concurrent canal dysfunction was associated with poor hearing outcome in SSHL (30). However, the pooled hearing recovery in the abnormal cVEMP response group was nearly half that of the recovery in the normal cVEMP response group, but this difference was still not statistically significant. This suggests that S + I lesions are not a critical variable in determining the extent of hearing improvement in SSHL. Further clinical data on the hearing outcomes of S + I lesions (by cVEMP tests) and U + S lesions (by oVEMP tests) are needed in order to draw more robust conclusions.

To the best of our knowledge, this is the first systematic review and meta-analysis to quantitatively summarize the prevalence of vestibulocochlear lesion patterns and to explore the prognostic role of vertigo in inner ear organ lesion patterns in SSHL. The results were extensive enough to be valid, including both pooled event rates within non-comparative studies and dichotomous data within comparative studies. In addition, the rule of omission was applied to assess and correct for publication bias in the sensitivity analysis.

There are several limitations that must be considered in the current systematic review. First, most of included studies were generally retrospective observational studies, which introduced publication bias and might not necessarily infer causation in our analysis. There was also a wide variety in the type of hearing loss, the degree of vertigo, and other accompany symptoms, as described in each study, which might reduce the reliability of our analysis. Third, the inter-study heterogeneity based on population differences was not evaluated using a meta-regression analysis. Therefore, the results should be interpreted with caution, and future well-designed studies are needed to investigate the causation of vestibular dysfunction in SSHL.

## Conclusion

Our overall associations indicated that patients are at a substantially increased risk of vestibular organ lesions following a diagnosis of SSHL, especially in the presence of vertigo. Nearly half of the patients suffered from vestibular dysfunction in SSHL, and LSC + S lesions might be a critical variable in determining the extent of hearing improvement in SSHL. Thus, we conclude that the focus should not be just on the cochlea but also on the diagnosis and treatment of the vestibular system in SSHL regardless of the presence of vertigo. Emphasis on vestibular function might also be of important significance in the clinical manifestation and prognosis of SSHL.

## Author Contributions

The literature was screened and methodological quality was assessed independently by HY and HL. HL drafted the manuscript. HY and HL performed the meta-analysis. All authors approved the final version and agree to be accountable for this work.

## Conflict of Interest Statement

The authors declare that the research was conducted in the absence of any commercial or financial relationships that could be construed as a potential conflict of interest.
